# Analysis of saponin composition and comparison of the antioxidant activity of various parts of the quinoa plant (*Chenopodium quinoa* Willd.)

**DOI:** 10.1002/fsn3.1358

**Published:** 2019-12-19

**Authors:** Jeong Gyu Lim, Hyun‐Mee Park, Ki Sun Yoon

**Affiliations:** ^1^ Department of Food and Nutrition Kyung Hee University Seoul Korea; ^2^ Advanced Analysis Center Korea Institute of Science and Technology Seoul Korea

**Keywords:** antioxidant capacity, quinoa plant, root, sapogenins, saponins, sprout

## Abstract

Quinoa plant is a valuable food crop because of its high nutritional and functional values. Total saponin content, sapogenins, polyphenol, and flavonoid contents and antioxidant activities were analyzed in various parts of quinoa plants, including sprout, seeds, bran, pericarp, leave, stem, and root. Quinoa seeds (QS) had significantly higher sapogenin content than quinoa stem (QT), quinoa leaves (QL), and quinoa roots (QR). Quinoa saponin was mainly composed of phytolaccagenic acid. Quinoa root (QR) had the highest amount of total saponin (13.39 g 100 g^−1^), followed by quinoa bran. The highest total phenolic content (30.96 mg GAE 100 g^−1^) and total flavonoid content (61.68 mg RE 100 g^−1^) were observed in quinoa root extract and 1‐month‐old sprout extract, respectively. Quinoa sprouts showed better antioxidant activity than fully grown parts of the quinoa plant. Overall, root and sprout had a higher antioxidant capacity compared to other parts of the quinoa plant, suggesting the potential use of quinoa root and sprout as a nutraceutical ingredient in the health food industry.

## Graphical abstract

1. A method for sapogenin analysis in extracts of various parts of the quinoa plant grown in Korea was developed and validated using GC‐MS/MS. 2. Total saponin, polyphenols, and flavonoids contents were the highest in quinoa roots among various parts of the quinoa plant. 3. Contents of three sapogenins were the highest in quinoa seed bran. 4. The functional aspect of quinoa sprout, bran, leaves, and roots was confirmed.

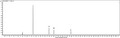

## 1 INTRODUCTION

Quinoa (*Chenopodium quinoa* Willd.) is a food crop that has been grown in the Andean region of Bolivia and Peru for the past 5,000–7,000 years. Recently, quinoa has been cultivated in places other than the Andean region, spreading all over the world. Quinoa is recognized as a substitute crop because of its survival in harsh environments. Besides, quinoa has been attracting attention not only due to its high nutritional value, but also due to its essential therapeutic compounds, such as saponins, phytosterols, squalene, and polyphenols (Alvarez‐Jubete, Wijngaard, Arendt, & Gallagher, 2010). Among these compounds, phenolic materials can act as antioxidants and eventually prevent many diseases (Gawlik‐Dziki et al., 2013). Moreover, extracts of quinoa seeds have shown higher contents of anthocyanins and polyphenols and higher antioxidant activities than amaranth seeds (Paśko et al., 2009).

Quinoa saponins are classified as triterpenoids mostly derived from oleanolic acid, hederagenin, phytolaccagenic acid, serjanic acid, and 3β, 23, 30‐trihydroxy olean‐12‐en‐28‐oic acid that is present in seeds, seed coats, fruits, and flowers of quinoa plant (Kuljanabhagavad, Thongphasuk, Chamulitrat, & Wink, 2008). Quinoa saponins are not toxic to humans (Zhu et al., 2002) and antifungal (Stuardo & San Martín, 2008), immunoadjuvant (Verza et al., 2012) and anti‐inflammatory (Yao, Yang, Shi, & Ren, 2014) activities of quinoa saponin have been reported. Saponin content in quinoa seed is affected by its growth stage and the environment. It has the highest content at the blooming stage. Its content then decreases at the stage of grain filling. The environment, such as water and salinity, can also affect its content (Solíz‐Guerrero, de Rodriguez, Rodríguez‐García, Angulo‐Sánchez, & Méndez‐Padilla, 2002; Zurita‐Silva, Fuentes, Zamora, Jacobsen, & Schwember, 2014). During the process of obtaining edible quinoa seeds, a washing process is carried out to reduce the bitter taste from saponin in the external layers of the seed coat after threshing (Fiallos‐Jurado et al., 2016). Traditionally, the vanillin‐sulfuric acid assay is widely used for measuring total saponin content. Sapogenin can be analyzed by GC‐MS after silylation using N, O‐Bis (trimethylsilyl) trifluoroacetamide (BSTFA). GC‐MS/MS analysis can be used to remove superfluous substances in the identification of quinoa sapogenin, making it a reliable tool in the analytical process (Mad, Sterk, Mittelbach, & Rechberger, 2006).

Since various parts of the quinoa plant are thought to be worthless, many studies have been focused on nutritional and functional values of quinoa seeds only. Most of the processed quinoa products are made from quinoa seeds or whole grain. These products are used as a breakfast food for children, animal feed, medical purpose, or other industrial applications (FAO, 2013). Few studies have been conducted on quinoa leaves extract (Fiallos‐Jurado et al., 2016; Gawlik‐Dziki et al., 2013), and very little information exists in total saponin content of various parts of the quinoa plant, including leaves and root.

Therefore, the objective of this study was to analyze saponin content, including their sapogenins in extracts of quinoa seed and various parts of quinoa plant grown in Korea using GC‐MS/MS for their utilization as an economical food source. Besides, antioxidant activities of quinoa seeds, bran, pericarp (seed coats), leave, stem, and root were evaluated according to their growth stage in order to know how the growing stage influences the content of these compounds in quinoa plant. This study is the first one to compare total saponin, sapogenins, polyphenol, and flavonoid contents and antioxidant activities among various parts of the quinoa plant.

## 2 MATERIALS AND METHODS

### 2.1 Standard preparation and reagents

Oleanolic acid, hederagenin, and cholesterol were purchased from Sigma‐Aldrich. Phytolaccagenic acid was purchased from Carbosynth. Oleanolic acid and phytolaccagenic acid were dissolved in ethyl acetate while hederagenin was dissolved in methanol. Then, each standard was prepared at a concentration of 1 mg/ml. Ammonia water, *N*,*O*‐Bis(trimethylsilyl) trifluoroacetamide (BSTFA) with 1% trimethylchlorosilane (TMCS), anhydrous pyridine, ethanol, rutin, gallic acid, folin–ciocalteu reagent, 2,2‐diphenyl‐1‐picrylhydrazyl, 2,2′‐azino‐bis(3‐ethylbenzothiazoline‐6‐sulfonic acid) diammonium salt, phosphate buffer (pH 6.6), potassium hexacyanoferrate(III), trichloroacetic acid, and iron(III) chloride were purchased from Sigma‐Aldrich. Sodium sulfate anhydrous, hydrochloric acid, sodium carbonate, sodium hydroxide, petroleum ether, glacial acetic acid, and sulfuric acid were purchased from Daejung Chemicals & Metals. Ethyl acetate and methanol were purchased from J. T Baker.

### 2.2 Sample preparation

Quinoa (*C. quinoa* Willd.) used in this work was supplied from the Hongcheon River Farming Union (Hongcheon, Korea). It was planted in March and harvested in August 2017. Extracts of samples were produced using the method of Carciochi, Manrique, and Dimitrov (2015) with a slight modification. All quinoa samples were ground with a blender and passed through 1.14 mm mesh. Then, 10 g of each quinoa sample powder was dissolved in 200 ml of 80% ethanol. The mixture was extracted at 60°C in a water bath for 1 hr. After that, the extract was centrifuged at 36288xg (1736MGR, GYROZEN) for 10 min. The supernatant was filtered through a filter paper (Whatman No. 1) and concentrated under reduced pressure (GMG‐2000, EYELA). The extract was then freeze‐dried (FDB‐5502, Operon Co., Ltd.) and stored at −80°C freezer until it was used. Extracted samples included 1‐m‐old sprout (1SE), 3‐m‐old sprout (3SE), quinoa seed (QSE), quinoa seed brans (QBE), quinoa seed pericarp (QPE), quinoa stem (QTE), quinoa leaves (QLE), and quinoa root (QRE). The extract of each sample was used to measure amounts of polyphenol, flavonoid contents, and antioxidant activities of various parts of quinoa plants.

### 2.3 Quantification of total saponin and sapogenins

#### 2.3.1 Extraction of saponin and sapogenin and derivatization

Analysis of quinoa sapogenins (oleanolic acid, hederagenin, and phytolaccgenic acid) was performed based on the method of Gómez‐Caravaca, Iafelice, Verardo, Marconi, and Caboni (2014) with some modifications. The quinoa powder was defatted by petroleum ether. Then, 0.1 g of defatted sample with 50 μl of internal standard (IS) was refluxed with 3 N hydrochloric acids (methanolic solution) for 3.5 hr. The hydrolyzated sample was cooled at room temperature and neutralized with ammonia water. The hydrolysate was concentrated under reduced pressure (GMG‐2000, EYELA). The concentrate was then dissolved in 4 ml of distilled water and liquid–liquid partitioned with 3 ml of ethyl acetate. After repeating this step three times, ethyl acetate fractions were combined and filtered with sodium sulfate anhydrous (Saponin extract). For gas chromatography analysis, the final extract was dried in a nitrogen stream. After that, 200 μl of *N*,*O*‐Bis(trimethylsilyl) trifluoroacetamide (BSTFA) with 1% trimethylchlorosilane (TMCS) and 200 μl of anhydrous pyridine were added for derivatization. The mixture was heated at 70°C for 60 min. Finally, 1 μl of the derivatized extract was injected into a gas chromatography.

#### 2.3.2 GC‐MS/MS analysis

GC‐MS/MS analysis was performed using an Agilent 7890B combined with Agilent 7010B Triple Quadrupole GC/MS System (Agilent Technologies). The column used for the analysis in this work was an Agilent J&W GC column DB‐5MS (30 m × 0.25 mm × 0.25 μm). One μl of the derivatized sample was injected into the GC at a 10:1 split mode. The temperature at injection was 300°C. GC oven condition was maintained at 200°C for 3 min. It was then increased to 310°C at a rate of 15°C per min and held for 20 min. The total analysis time was 31 min, and the solvent delay was 10 min. Mass analysis was performed using electron impact ionization mode. All data were obtained using an electron multiplier with a gain factor of 10. Total mass spectra of analytes were obtained from 50 to 800 *m*/*z* at 70 eV. Mass ion source temperature and transfer line temperature were set at 250 and 300°C, respectively. For optimization conditions of multiple reaction monitoring (MRM) mode, a product ion of precursor ions was first searched under collision energy conditions at 10–40 eV. Quantitative analysis was performed by selecting quantitative and qualitative ions from optimized product ion. Analytes were identified using qualitative ion and retention time. Their contents in samples were determined using quantitative ion. All mass spectrometer analysis conditions were determined using standards for all analytes. Established retention time and optimized target ion information for each substance are shown in Table 1.

**Table 1:** Retention time and optimized target ions of analytes

Compounds	Retention time (min)	MRM transitions/(collision energy)	
		Quantifier ion (*m*/*z*)	Qualifier ion (*m*/*z*)
Oleanolic acid	15.44	320.3 > 203/(10)	482.5 > 190.2/(20)
Hederagenin	16.55	320.3 > 203/(10)	570.6 > 190.2/(10)
Phytolaccagenic acid	20.39	364.3 > 187/(15)	614.6 > 148.1/(10)
Cholesterol (I.S)	11.86	329.4 > 95.1/(20)	368.4 > 145.1/(20)

#### 2.3.3 Validation of the analytical method for sapogenin analysis

Validation was conducted to guarantee the quality of the method. Calibration curves were obtained by diluting each sapogenin standard (oleanolic acid, hederagenin, and phytolaccagenic acid). The accuracy and precision were determined by spiking sapogenin standards to the quinoa leave matrix at levels of 0.1 μg/g and 1 μg/g. The average concentration observed in the blank matrix was subtracted from all spiked matrix. All analyses were repeated four times. The linearity was evaluated by the correlation coefficient (*R* ^2^) of the calibration curve. The matrix was spiked at the lowest level (0.1 μg/g) of standards to degermine limits of detection (LOD) and limits of quantification (LOQ) of standards. Cholesterol was added as an internal standard in all validation procedures to correct for the loss of analyte in the extraction process, matrix effect, and degree of derivatization. LOD was calculated using the *t*_99_ (*t* value at 99% confidence of *n *− 1 degrees of freedom) based on the standard deviation of the response and the slope of the calibration curve. LOQ was calculated as ten times the standard deviation, considering the slope used to calculate LOD.

#### 2.3.4 Total saponin content

Total saponin content was determined through spectrophotometry, as described by Medina‐Meza, Aluwi, Saunders, and Ganjyal (2016). Briefly, 0.25 ml of saponin extract of each sample solution was added to 1 ml of reagent mixture (glacial acetic acid/sulfuric acid 1:1 v/v). The mixture was vortexed and reacted at 60°C in a water bath for 30 min and then cooled. The absorbance of the sample was measured at a wavelength of 527 nm using a spectrophotometer (Multiskan GO, Thermo Scientific). Oleanolic acid was used as a standard (0–1,000 μg/ml). Total saponin content was expressed as g 100 g^−1^ of oleanolic acid equivalents.

### 2.4 Polyphenol and flavonoid contents

Total phenolic content (TPC) was measured according to the modified method of Khatiwora, Adsul, Kulkarni, Deshpande, and Kashalkar (2010). In brief, 0.5 ml of 50% folin‐ciocalteu reagent (Sigma‐Aldrich) and 1 ml of 10% sodium carbonate were added to 1 ml of an extract of each sample. The mixture was allowed to stand at room temperature for 60 min in a dark place, and the absorbance was measured at a wavelength of 725 nm using a spectrophotometer. Gallic acid was used as a standard material at a concentration range of 0–100 μg/ml. Total phenolic content (TPC) was expressed as mg of gallic acid equivalents (GAE) 100 g^−1^.

Total flavonoid content (TFC) was measured according to the modified method of Abeysinghe et al. (2007). Briefly, 1 ml of diethylene glycol was added to 1 ml of an extract of each sample. Subsequently, 0.1 ml of 4 N sodium hydroxide was added to the mixture, followed by reaction at 40°C for 60 min. Absorbance was then measured at 420 nm using a spectrophotometer. Rutin was used as a standard material at a concentration range of 0–100 μg/ml. Total flavonoid content was expressed as mg of rutin equivalents (RE) 100 g^−1^.

### 2.5 Antioxidant activity

#### 2.5.1 2,2‐Diphenyl‐1‐picrylhydrazyl (DPPH) free radical scavenging assay

DPPH free radical scavenging assay of quinoa extracts was performed according to the method of Afify and Hassan (2016) with some modifications. Briefly, 0.5 ml of DPPH solution (0.2 mM in ethanol) was added to 0.5 ml of an extract of each sample. After standing at room temperature for 30 min, the absorbance was measured at a wavelength of 517 nm using a spectrophotometer. The free radical scavenging activity of each sample solution was compared to a blank sample. IC_50_ (the concentration required to inhibit 50% of the radical formation) of the DPPH radical scavenging activity was also calculated.

#### 2.5.2 2,2′‐Azino‐bis (3‐ethylbenzothiazoline‐6‐sulfonic acid) diammonium salt‐free radical scavenging assay

ABTS free radical scavenging assay of quinoa extract was performed according to the method of Shalaby and Shanab (2013). Briefly, 7 mM ABTS solution was mixed with 2.45 mM potassium persulfate (1:1, v/v). The mixture was left at room temperature for 16 hr. The ABTS solution was then diluted with ethanol until the absorbance reached 0.70 ± 0.02 at 734 nm. After mixing 50 μl of the extract solution with 950 μl of ABTS solution and incubating at room temperature for 15 min, the absorbance of the mixture was measured at 734 nm using a spectrophotometer. The free radical scavenging activity of each sample solution was compared to a blank sample. IC50 (the concentration required to inhibit 50% of the radical formation) of the ABTS radical scavenging activity was also calculated.

#### 2.5.3 Reducing power

Reducing power of quinoa extract was measured according to the modified method of Lue et al. (2010). Briefly, 1.25 ml of 0.2 M phosphate buffer (pH 6.6) and 1.25 ml of 30 mM potassium hexacyanoferrate (III) were added to 0.5 ml of the extract of each sample and incubated at 68°C for 20 min. Then, 1.25 ml of 0.6 M trichloroacetic acid was added, and the mixture was centrifuged at 4032xg for 10 min. After collecting the supernatant, 1 ml of distilled water and 0.2 ml of iron (III) chloride were added to 1 ml of the supernatant and left at room temperature for 15 min. The absorbance of the final test solution was measured at 700 nm using a spectrophotometer. EC50 (the concentration needed to cause 50% of the antioxidant effect) of the reducing power was also calculated.

### 2.6 Statistical analysis

All experiments were repeated three times. All data were analyzed using Statistical Analysis Systems SAS, version 9.3 (SAS Institute, Cary, NC, USA). One‐way analysis of variance (ANOVA) was performed, and a significant difference was determined with Duncan's multiple range test at *p* < .05.

## 3 RESULTS AND DISCUSSION

### 3.1 Validation of the analytical method for sapogenin analysis

Within the concentration range in this work, all correlation coefficients were above 0.99 (Table 2). The recovery of spiked analyte was measured to determine the accuracy of the method, comparing blank samples with known amounts of spiked standards. The precision was expressed as a relative standard deviation (RSD). The accuracy of oleanolic acid (OA) was 91.24%–92.91%, and the % RSD was 3.18–3.26. The accuracy of hederagenin (HD) was 82.6%–87.43% and the % RSD of 2.76–3.37. In the case of phytolaccagenic acid (PA), the accuracy and % RSD were 85.66%–103.72% and 2.68–2.76, respectively. The limit of detection (LOD) of OA, HD, and PA was 0.003, 0.003, and 0.013 μg/g, respectively. The limit of quantification (LOQ) of OA, HD, and PA was 0.007, 0.008, and 0.028 μg/g, respectively.

**Table 2:** Validation of the analytical method for sapogenin analysis

Compound	Calibration curve	Spiked level	Recovery %	RSD	LOD (μg/g)	LOQ (μg/g)
Oleanolic acid	*y* = 3.99*ⅹ* − 0.2097 (*R* ^2^ = .993)	0.1 μg/g	91.24	3.18	0.003	0.007
		1 μg/g	92.91	3.26		
Hederagenin	*y* = 3.4719*ⅹ* − 0.1708 (*R* ^2^ = .994)	0.1 μg/g	82.6	2.76	0.003	0.008
		1 μg/g	87.43	3.37		
Phytolaccagenic acid	*y* = 0.8079*ⅹ* − 0.0468 (*R* ^2^ = .998)	0.1 μg/g	85.66	2.68	0.013	0.028
		1 μg/g	103.72	2.76		

Abbreviations: LOD, limits of detection; LOQ, limits of quantification.

### 3.2 Contents of sapogenins and total saponin

**Figure 1:**
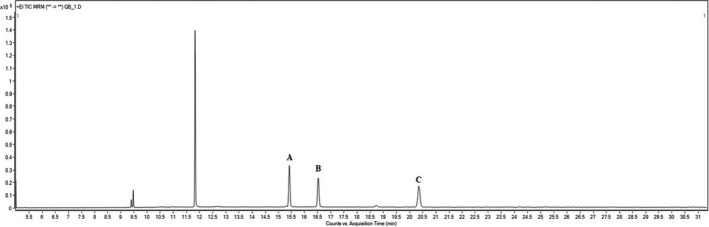
Chromatogram of GC‐MS/MS MRM analysis of sapogenins in quinoa samples. (A) Oleanolic acid‐TMS, (B) hederagenin‐TMS, (C) phytolaccagenic acid‐TMS

Major aglycones of quinoa saponin are oleanolic acid (OA), hederagenin (HD), and phytolaccagenic acid (PA). They determine the structural properties of quinoa saponin. Serjanic acid is also known as an aglycone of quinoa saponin. However, its relative amount in the quinoa plant is low compared to other aglycones (Ruiz et al., 2017). In the present study, sapogenins in the various parts of quinoa plants were quantified using GC‐MS/MS. The chromatogram of quinoa sapogenins is shown in Figure 1. To quantitate sapogenins, fragmentation patterns of trimethylsilylated (TMS) OA, HD, and PA (Table 3) were analyzed using standards, and the optimized target ion was set by comparing the sensitivity of a product ion in the mass spectrometer. OA‐TMS and HD‐TMS showed relatively high reactivity at 203 *m*/*z*. The product ion was selected as the optimal target ion for quantification. In PA‐TMS, product ion presented at 187 *m*/*z* was selected for appropriate PA‐TMS quantification. As a result of quantitating quinoa sapogenins with the above optimum conditions, there was no significant difference in the amount of sapogenins between 1‐m‐old sprout (1S) and 3‐m‐old sprout (3S), indicating no change in the amount of sapogenins according to the growth stage (Table 4). Contents of OA, HD, and PA in quinoa seeds (QS) were 0.301, 0.300, and 1.650 mg/g, respectively. Quinoa seeds had significantly higher sapogenin content than quinoa stem (QT), quinoa leaves (QL), and quinoa roots (QR). Quinoa bran (QB) had the highest amounts of OA, HD, and PA, while quinoa leaves (QL) had the lowest sapogenin contents. In this work, PA was the most commonly found sapogenin in the various parts of quinoa plant grown in Korea, which is consistent with results of a previous study on sapogenin content in quinoa seeds grown in the United States (Medina‐Meza et al., 2016). On the other hand, Mastebroek, Limburg, Gilles, and Marvin (2000) found that hederagenin (HD) was the major sapogenin found in leaves and oleanolic acid (OA) was in seeds.

**Table 3:** Trimethylsilylated (TMS) oleanolic acid, hederagenin, and phytolaccagenic acid in GC‐MS/MS fragmentation patterns

Compound	Structure		
		MS 1	MS 2
Oleanolic acid (OA)‐TMS	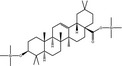	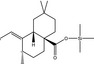	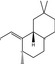
	*m*/*z* 600	*m*/*z* 320	*m*/*z* 203
Hederagenin (HD)‐TMS	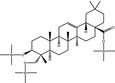	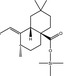	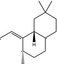
	*m*/*z* 688	*m*/*z* 320	*m*/*z* 203
Phytolaccagenic acid (PA)‐TMS	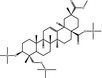	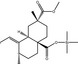	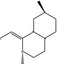
	*m*/*z* 732	*m*/*z* 364	*m*/*z* 187

**Table 4:** Amounts of three major sapogenins^1^ and total saponin^2^ in various parts of the quinoa plant

	1S	3S	QS	QB	QP	QT	QL	QR
OA	^AB^0.007 ± 0.000^d^	^AB^0.006 ± 0.001^d^	^B^0.301 ± 0.013^c^	^B^7.449 ± 0.189^a^	^B^0.475 ± 0.025^b^	^A^0.025 ± 0.004^d^	^A^<0.001^d^	^A^0.003 ± 0.000^d^
HD	^B^0.005 ± 0.000^c^	^A^0.001 ± 0.000^c^	^B^0.300 ± 0.041^b^	^B^7.501 ± 0.311^a^	^B^0.516 ± 0.044^b^	^B^0.008 ± 0.002^c^	^B^<0.001^c^	^B^0.001 ± 0.000^c^
PA	^A^0.009 ± 0.002^c^	^B^0.005 ± 0.001^c^	^A^1.650 ± 0.254^bc^	^A^27.455 ± 2.019^a^	^A^2.410 ± 0.116^b^	^A^0.040 ± 0.011^c^	^A^<0.001^c^	^A^0.003 ± 0.001^c^
TS	1.21 ± 0.08^de^	1.29 ± 0.06^de^	1.26 ± 0.03^de^	8.34 ± 0.38^b^	1.60 ± 0.02^d^	3.67 ± 0.30^c^	0.97 ± 0.02^e^	13.39 ± 0.22^a^

Note

Values are expressed as mean ± standard deviation.

Abbreviations: 1S, 1‐m sprout; 3S, 3‐m sprout; HD, hederagenin; OA, oleanolic acid; PA, phytolaccagenic acid; QB, quinoa bran; QL, quinoa leaves; QP, quinoa pericarp; QR, quinoa root; QS, quinoa seeds; QT, quinoa steam; TS, total saponin.

^a‐e^Means values with different letters in the same row are significantly different by Duncan's multiple range test at *p* < .05.

^A‐B^Means values with different letters in the same column are significantly different by Duncan's multiple range test at *p* < .05.

^1^ Unit: mg/g.

^2^ Unit: g 100 g^−1^.

Total saponin (TS) content of quinoa was also determined by measuring purple color produced by the reaction of acid and saponin. In the present work, an increase of total saponin amount was not observed according to the growth stage of sprouts from 1 m to 3 m. The total saponin content of quinoa seeds was 1.26% (1.26 g 100 g^−1^), which was lower than that in other parts of the quinoa plant except for quinoa leaves (0.97 g 100 g^−1^). Quinoa root (QR) had the highest amount of total saponin (13.39 g 100 g^−1^), followed by quinoa bran (QB), quinoa stem (QS), quinoa seed pericarp (QP), and quinoa leaves (QL). An amount of 0.9 g 100 g^−1^ dry leaf matter in some quinoa genotypes was also reported (Gupta & Wagle, 1988). It is important to notice that there is not much information about saponin content in other parts of the quinoa plant. Since most of the previous studies evaluated the saponin content of quinoa grain or seeds only, simple comparison for the amount of saponin in other parts of the quinoa plant with other studies is impossible. Besides, saponins decreased if quinoa samples were exposed to drought and saline regimens (Gómez‐Caravaca et al., 2012). Moreover, compositions and amounts of saponin in quinoa seeds were not always the same because bran and pericarps of seeds were continuously removed in the process of harvesting and making edible quinoa seed (Gómez‐Caravaca et al., 2014) The total saponin content of quinoa grown in Washington State ranged from 3.81 to 27.1 mg/g (Medina‐Meza et al., 2016; Nickel, Spanier, Botelho, Gularte, & Helbig, 2016), which showed that the washing process under running water reduced the content of saponins. However, the saponin content remained similar after the processes of cooking at atmospheric pressure, under pressure, and toasting.

### 3.3 Total polyphenol and flavonoid

Polyphenols are naturally present in plants and have antioxidant potential, that is, the ability to eliminate free radicals (Shalaby & Shanab, 2013). Their antioxidant capacity depends on the number and arrangement of hydroxyl groups in the phenolic material that can decrease the progress of oxidation by transferring hydrogen atoms to radicals (Shahidi & Ambigaipalan, 2015). Total polyphenol and flavonoid contents were evaluated, and their amounts in various parts of the quinoa plant are shown in Table 5. Total phenolic content (TPC) of quinoa root extract (QRE) was 30.96 mg GAE 100 g^−1^, which is the highest amount among the extracted sample of quinoa plant, followed by 1‐m sprout (1SE:28.47 mg GAE 100 g^−1^) and 3‐m sprout (3SE:24.02 mg GAE 100 g^−1^). TPC in quinoa sprout decreased significantly when quinoa sprout grew during 3 m of the growth period. TPC of quinoa seed extract (QSE) was 14.37 mg GAE 100 g^−1^. A similar result was reported (Park, Lee, Kim, & Yoon, 2017) that the TPC of quinoa seed cultivated in Korea was 14.50 mg GAE 100 g^−1^. Compared to quinoa seed extract (QSE), higher polyphenol contents were observed in quinoa bran extract (QPE), quinoa leaves extract (QLE), and quinoa root extract (QRE). Besides, the highest total flavonoid content (TFC) was observed in 1SE (61. 68 mg RE 100 g^−1^) and decreased significantly in the 3SE (50.89 mg RE 100 g^−1^), as shown in the results of TPC. However, TFC of quinoa seed extract (QSE: 45.88 mg RE 100 g^−1^) was significantly lower than those of 1SE and 3SE. The highest TFC was also observed in quinoa root extract (54.14 mg 100 g^−1^), followed by QLE (51.29 mg 100 g^−1^), QSE (45.88 mg 100 g^–1^), QTE (42.07 mg 100 g^–1^), and QPE (26.80 mg 100 g^−1^). The lowest TPC and TFC were observed in quinoa bran extract (QBE). Overall, quinoa root showed the highest content of antioxidant materials, indicating that the antioxidant activity of quinoa root would be the best among the quinoa plant.

**Table 5:** Total polyphenol and flavonoid contents in the extracts of various part of the quinoa plant

	1SE	3SE	QSE	QBE	QPE	QTE	QLE	QRE
TPC^1^	28.47 ± 0.28^b^	24.02 ± 0.07^c^	14.37 ± 0.11^e^	9.05 ± 0.05^g^	18.39 ± 0.06^d^	11.73 ± 0.09^f^	18.09 ± 0.10^d^	30.96 ± 0.29^a^
TFC^2^	61.68 ± 1.38^a^	50.89 ± 2.24^c^	45.88 ± 1.40^d^	9.41 ± 0.23^g^	26.80 ± 1.11^f^	42.07 ± 0.83^e^	51.29 ± 1.21^bc^	54.14 ± 0.78^b^

Abbreviations: 1SE, 1‐m sprout extract; 3SE, 3‐m sprout extract; QBE, quinoa bran extract; QLE, quinoa leaves extract; QPE, quinoa pericarp extract; QRE, quinoa root extract; QSE, quinoa seeds extract; QTE, quinoa steam extract; TFC, total flavonoid content; TPC, total polyphenol content.

^a‐f^Means values with different letters in the same row are significantly different by Duncan's multiple range test at *p* < .05.

^1^ Unit: mg GAE 100 g^−1^ dry material.

^2^ Unit: mg RE 100 g^−1^ dry material.

### 3.4 Antioxidant activities of various parts of the quinoa plant

IC50 of DPPH and ABTS radical scavenging activity and EC50 of reducing the power of various parts of quinoa plant extract were measured to evaluate antioxidant activities of various parts of the quinoa plant extracts (Table 6). Relatively low IC50 values of DPPH radical scavenging assay related to higher antioxidant activity. QRE had IC50 value of 0.51 mg mL^‐1^, which was the lowest among various parts of quinoa plant. There was no significant difference among QRE (0.51mg mg mL^‐1^), 3m sprout (0.52 mg mL^‐1^), QLE (0.54 mg mL^‐1^), 1m sprout (0.55 mg mL^‐1^), and QSE (0.58 mg mL^‐1^), indicating that 1m old sprout has the same antioxidant capacity as quinoa seed extract (QSE). Similar results were also observed in ABTS free radical scavenging activity and reducing power. There are no significant differences among 1SE, 3SE, QSE, and QRE, where the lowest IC50 value (2.49 mg/ml) of ABTS was observed in 3‐m sprout (Table 6). Quinoa root extract (QRE) had a lower EC50 value of 2.03 mg/ml than those of QBE (8.73 mg/ml), QTE (4.56 mg/ml), QPE (4.42 mg/ml), QTE (4.56 mg/ml), and QLE (3.29 mg/ml). Overall, quinoa sprouts showed better antioxidant activity than fully grown quinoa stem, and leaves, indicating that quinoa sprout can be a healthy diet source like quinoa seed. Besides, various parts of quinoa showed different levels of antioxidant capacity. This result might be due to differences in their phenolic compound contents. Previous studies have also reported that quinoa sprouts have higher antioxidant capacity than a fully grown quinoa (Paśko et al., 2009). In previous studies, IC50 values of DPPH free radical scavenging activity of quinoa seeds cultivated in Chile (Miranda et al., 2014) were 0.4–3.7 mg/mg_._ It was 4.39 mg/ml for quinoa seeds grown in Brazil (Nickel et al., 2016). Both EC50 of ABTS free radical scavenging activity and reducing power of ethanolic extract from quinoa leaves (Gawlik‐Dziki et al., 2013) were 8 mg/ml_._ Compared with cultivated quinoa seeds studied in other countries, quinoa seeds cultivated in Korea generally showed better antioxidant activities. On the other hand, measured IC50 and EC50 values in the antioxidant assay were the lowest for quinoa root extract (QRE) among various parts of the quinoa plant in the present study. These results comprehensively suggest that quinoa roots might have good nutraceutical potential, and further application and usage as food sources must be studied.

**Table 6:** Antioxidant activities in the extracts of various part of the quinoa plant

	1SE	3SE	QSE	QBE	QPE	QTE	QLE	QRE
DPPH^1^	0.55 ± 0.00^d^	0.52 ± 0.01^d^	0.58 ± 0.00^d^	1.51 ± 0.03^b^	1.04 ± 0.11^c^	1.65 ± 0.02^a^	0.54 ± 0.01^d^	0.51 ± 0.00^d^
ABTS^1^	2.65 ± 0.07^e^	2.49 ± 0.12^e^	2.54 ± 0.03^e^	10.78 ± 0.05^a^	7.14 ± 0.30^b^	4.02 ± 0.11^c^	3.39 ± 0.13^d^	2.65 ± 0.03^e^
Reducing power^2^	1.46 ± 0.04^f^	2.71 ± 0.07^d^	1.54 ± 0.10^f^	8.73 ± 0.19^a^	4.42 ± 0.18^b^	4.56 ± 0.14^b^	3.29 ± 0.06^c^	2.03 ± 0.04^e^

Abbreviations: 1SE, 1‐m sprout extract; 3SE, 3‐m sprout extract; QBE, quinoa bran extract; QLE, quinoa leaves extract; QPE, quinoa pericarp extract; QRE, quinoa root extract; QSE, quinoa seeds extract; QTE, quinoa steam extract.

^a‐f^Means values with different letters in the same row are significantly different by Duncan's multiple range test at *p* < .05.

^1^ IC50; The concentration required to inhibit 50% of the radical formation (mg/ml).

^2^ EC50; The concentration needed to cause 50% of the antioxidant effect (mg/ml).

## 4 CONCLUSION

The presence of sapogenin, total saponin, and antioxidant capacities of 1‐m and 3‐m quinoa sprouts and other parts of fully grown quinoa plants were confirmed in this study. Besides, a method for simultaneously analyzing oleanolic acid, hederagenin, and phytolaccagenic acid in various parts of quinoa plants was developed and validated using GC‐MS/MS. This analytical method showed lower detection limits than previous studies. Besides, its accuracy, repeatability, and high linearity were appropriate for analyzing sapogenins in quinoa. Amounts of oleanolic acid, hederagenin, and phytolaccagenic acid were different according to various parts of quinoa, including sprouts and fully grown parts of the quinoa plant. Contents of three sapogenins were the highest in quinoa seed bran but the lowest in quinoa leaves and roots. However, total saponin content was the highest in quinoa roots, suggesting that saponins in quinoa roots were mainly composed of other sapogenins that were not analyzed in this study. Both polyphenols and flavonoids contents were the highest in quinoa roots but the lowest in quinoa bran. Quinoa root contains the highest amount of total saponin with the highest antioxidant capacity, similar to quinoa seeds. This study is the first time that the saponin content of quinoa root has been quantitated. Since only quinoa seeds are commercially distributed and other parts of the quinoa plant are being discarded, sprout, quinoa leaves, quinoa bran, and quinoa roots might have good nutraceutical potential, and further application and usage as food sources need to be investigated in the future study.

## CONFLICT OF INTEREST

The authors declare that they have no conflict of interests.

## ETHICAL APPROVAL

The human and animal testing was unnecessary in the current study.
